# SEC Separation of Polysaccharides Using Macroporous Spherical Silica Gel as a Stationary Phase

**DOI:** 10.1007/s10337-018-3582-5

**Published:** 2018-08-06

**Authors:** Tomasz Krawczyk, Mariusz Zalewski, Anna Janeta, Paweł Hodurek

**Affiliations:** 1 Department of Chemical Organic Technology and Petrochemistry, Faculty of Chemistry, Silesian University of Technology, ul. Krzywoustego 4, 44-100 Gliwice, Poland; 2 Wroclaw Medical University, Wybrzeże L. Pasteura 1, 50-367 Wrocław, Poland

**Keywords:** Size-exclusion chromatography (SEC), Partition chromatography, Silica gel, Polysaccharides, Dextran, Alginic acid

## Abstract

**Abstract:**

Meso- and macroporous spherical silica gels of pore sizes in the range of 60–1000 Å and 40–75 µm particle size were investigated as a stationary phase for the separation and purification of polysaccharides and poly(ethylene glycols) (PEGs) of various MWs using an aqueous mobile phase. Sephadex and Bio-Gel were used for comparison as the most common stationary phases for similar purposes. The separation of dextrans of a mean MW = 31 kDa from small molecules (NaCl) was possible with SiO_2_ with a pore size of 60–300 Å, but the observed efficiencies of a column of the same size were lower comparing with Sephadex or Bio-Gel. In the case of oxidized alginic acid only SiO_2_ of the 60 Å pore size was suitable, while Sephadex, Bio-Gel and other investigated silicas were not efficient. Sephadex and 300–1000 Å SiO_2_ offered the possibility of dividing dextrans with MW within the range of 1 MDa–10 kDa into fractions of various MWs, while Bio-Gel and 60 Å SiO_2_ were not suitable. The investigated silica gels strongly adsorbed PEGs of MW 2–20 kDa. The amount adsorbed decreased with the increase of pore size and they were not useful as a stationary phase for this class of polymers. An advantage of SiO_2_ of the investigated particle size was a very low back pressure comparing with Sephadex. A considerably lower price of silica offers time- and cost-efficient separation of polysaccharides.

**Graphical Abstract:**

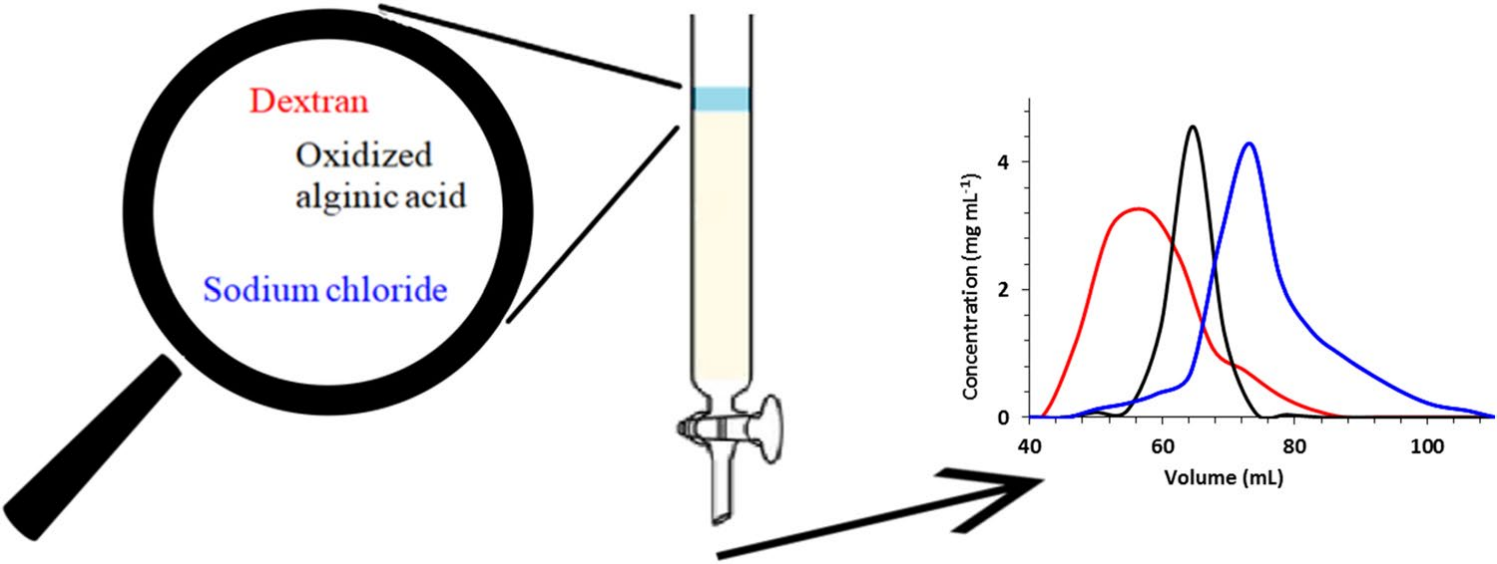

## Introduction

Polysaccharides and other glycans (like glycosamineglycans) are important classes of natural polymers. In plants, fungi, and animals they act as—among other things—an energy source, a part of cell walls or of the exoskeleton structure [1]. Some of them, such as cellulose, are an important feedstock in paper and textile industry. Hyaluronic acid (HA) and dextran are widely used in medicine and cosmetics [2, 3]. Due to numerous functional groups, polysaccharides are suitable as a scaffold for synthesis of functional materials, such as catalyst supports [4], drug carriers [5], or imaging probes [6].

Polysaccharides (and in general glycans) are obtained from natural sources using various isolation and purification techniques. For example, cellulose is obtained from wood biomass in the oxidative delignification process by acetic acid and hydrogen peroxide with a catalytic amount of sulfuric acid [7]. HA is obtained from rooster combs by extraction with sodium acetate and precipitation with an ethanol solution [8]. Dextran is now synthesized by lactic acid bacteria, such as *Leuconostoc mesenteroides* using sucrose [9]. During preliminary purification polysaccharides can be precipitated from their aqueous solution with organic solvents, such as acetone, acetonitrile or ethanol, sometimes with the aid of sodium chloride [10, 11]. The disadvantage of this method is the possibility of co-precipitation of impurities.

For biochemical and medicine-related applications it is also important to separate a particular fraction of a polysaccharide with a suitable MW. For example, 200–2000 kDa fractions of inulin, β-glucan, levan and HA are usually used for pharmaceutical-related purposes [7, 12, 13]. One of the reasons is MW-dependent properties of polysaccharides. For instance, low molecular weight HA can activate microphages in a classically activated-like state, while high molecular HA induces an alternatively activated-like state [12]. Therefore, methods of purification from natural sources and separation of polysaccharides into fractions of a particular MW suitable for analytical and preparative purposes are necessary. The most commonly used methods are: capillary electrophoresis (CE) [14, 15], combination of CE and HPLC [16], nanofiltration [17, 18], and size-exclusion chromatography (SEC).

In SEC the separation of substances is based on the hydrodynamic volumes of the analytes [19]. There are a lot of commercial columns on the market, mostly based on an acrylate polymer matrix [20]. The stationary phase in SEC is a porous three-dimensional network that the solute molecules can penetrate [19]. Partition between the pores and the mobile phase occurs depending on the hydrodynamic volume of the separated substances. Substances with a smaller hydrodynamic volume have a higher probability of getting into the bead pore [21]. The general rule is: the bigger the molar mass of the molecule is the shorter its retention time. Because the retention can be increased by undesired phenomena such as adsorption or enthalpic partition (absorption) it is important to prevent polymer-packing interactions in the routine SEC [22].

Silica gel is one of the oldest and widely available chromatographic beads. It is mainly used as an adsorption beads for normal-phase chromatography [23]. The adsorption capacity is mainly dependent on oxygen atoms, bonded to silicon atoms. A very good source of protons, like water, methanol or another auto-protolysing liquid, is able to completely deactivate silica gel as a normal-phase bead due to protonation of the terminal oxygens in silica gel. This is why a bare silica gel column in HPLC is 100 times less lasting than modified silica gel, i.e., the C_18_ group for RP or –diol for NP. Not all adsorption properties are destroyed this way. If pH of the water solution is above 6.0, negative charges may occurs on the bead and there will be a strong electrostatic force to positively charged molecules or groups in the (macro)molecule. This is why there are adsorption phenomena (sometimes irreversible) with proteins with higher pI (i.e., lysozyme) [24]. For proteins there are also other phenomena, like stability of the structure, when such a rigid bead is used. This is why silica gel can be used as a SEC bead for non-charged or slightly negatively charged macromolecules with a flexible structure, like polysaccharides, or non-positively charged glycans or other water-soluble polymers, like poly(ethylene glycol) (PEG) [18, 23–25].

For the purpose of separation of polysaccharide several types of silica was investigated. Earlier works [28] showed a possibility of fractioning dextrans of average MW 40 kDa into low and high fraction (25 and 70 kDa respectively) using mesoporous silica of particle size 200–500 micron. Bare silica was also investigated in HPLC mode (particle size 5 micron) [29] and SEC properties was showed for dextrans of MW between 5 and 100 kDa when pore size of the bed were 100–250 Å. Interestingly, a RP column with large pores (300 Å) was also suitable for purification and fractioning dextrans of MW 6–600 kDa if logMW difference was equal to 1 [30].

Commonly, SEC of polysaccharides is most often performed using polymer-based stationary phases. The focus of this study was to investigate the possibility of purification and separation of polysaccharides on a semi- or preparative scale with bare silica gel of wide range of pore size (60–1000 Å) and moderate particle size 40–75 micron. Two types of polysaccharides and PEGs were selected as model compounds. Comparison with other commonly used polymeric stationary phases was also included.

## Experimental

### Materials

Silica gels 40–75 µm, 300 Å, Part No.: S10030M; 40–75 µm, 500 Å, Part No.: S10030P; 40–75 µm, 1000 Å, Part No.: S10030P; were purchased from SiliCycle Inc. (Quebec city, Canada). Silica gel 60 Å, NaIO_4_ of the reagent grade purity and dextran of the biochemistry grade (low fraction ≈ 31 kDa) was delivered by Acros Organics (Geel, Belgium). Alginic acid (from brown algae) was purchased from Sigma-Aldrich (Steinheim, Germany). Narrow molar mass dispersity dextrans of MW 25, 50, 150 and 670 kDa and dextran from *Leuconostoc* spp. (fraction 450–650 kDa) were delivered by Sigma-Aldrich (St. Louis, MO). Poli(ethylene glycol) 2000 and 20000 were delivered by Fluka (Buch, Switzerland), while PEG 6000 was delivered by Schuchardt (Munich, Germany). Sephadex G-150 (40–120 µm) was purchased from Pharmacia Fine Chemicals (Uppsala, Sweden), while Bio-Gel P-2 Gel from Bio-Rad (Herkules, CA).

### Instruments

HPLC-SEC analyses were performed with a Waters 600E chromatograph equipped with RID (Knauer K-2301 or Waters 410), a Waters 717plus autosampler, a Waters Ultrahydrogel 2000 column (7.8 × 300 mm), and a Waters Ultrahydrogel 6 × 40 mm Guard Column. Pure water delivered at 0.8 mL min^−1^ was used as a mobile phase.

The low-pressure SEC system consisted of a glass column (20 × 400 mm) with sintered glass disc and stopcock with PTFE key.

## Methods

### Oxidation of Alginic Acid

A sample of water-soluble polysaccharide containing –COOH functionalities was prepared from native alginic acid by oxidation with NaIO_4_. Such procedure do not lead to significant polymer degradation [31]. A portion of alginic acid (200 mg) was dispersed in 20 mL of water and a solution of NaIO_4_ (200 mg in 5 mL water) was added. The mixture was left stirring overnight at room temperature. The solution was concentrated with rotary evaporator to 2 mL, and 50 mL acetone was added to precipitate the polymer which was separated then by centrifugation. The residue was washed with acetone, dissolved again in a small portion of water (5 mL), and precipitated again, centrifuged, washed with acetone, and dried.

### Preparation of Stationary Phases

A portion of 35 g of SiO_2_ was added to boiling water (300 mL) and left to cool down to room temperature. Such treatment allowed reduction of absorption properties of silicas. The rationale behind this is that the silicas are not fully surface-hydrated and the hydration process is typically slow at room temperature [25]. After decantation fresh water was added and the slurry was transferred into a glass column (20 × 400 mm). A 0.5-cm layer of sand was placed below, and above the silica bed. Biogel (25 g) was mixed with 300 mL of water and left overnight to swell, while Sephadex (2.5 g) was left for 3 days in 200 mL of water. The swelled phases were transferred into the column. In each case the height of the bed was 25 cm. The columns were packed by simple sedimentation in each case and were conditioned with 100 mL of water. Samples of the investigated substances (25–75 mg) were dissolved in water (1 mL) and pipetted carefully on the top of sand layer. Eluates were collected in 2–5 mL portions and analyzed using the HPLC system.

The critical overlap concentration c* [21] was calculated according to the equation below for dextrans and PEGs used.

$$c^*=\frac{{{M_{\text{w}}}}}{{\left( {\frac{{4\pi }}{3} \cdot {R_{\text{g}}}^{3}} \right){N_{\text{A}}}}}$$

The radius of gyration (*R*_g_) data were taken from [32]. For dextran of *M*_w_ = 506, 334, and 132 kDa *R*_g_ = 21, 19 and 12 nm respectively which translates into the critical overlap concentration equal to 22, 19 and 31 g L^−1^. The concentrations of samples were 25–150 g L^−1^ while the highest recorded concentration of dextran in eluate stream was 4 g L^−1^ (Fig. 4). In the case of PEG 2000 *R*_g_ = 1.2 nm [33] which gives c* = 460 g L^−1^. Taking into account that the sample was pipetted on the layer of wet sand and therefore entered the column diluted approximately by half, the concentrations used were reasonable but should not be further increased.

### Adsorption

Solutions of PEG 2000, 6000, 20000 and dextran 31 kDa of 1.5; 3; and 7.5 mg mL^−1^ were prepared by dissolving corresponding amounts of polymers in 100 mL of water in a volumetric flask. 200 mg of silica was weighted in centrifugal tubes (2 mL) and 1.5 mL of the solution was added. The mixtures were shaken for 30 min at room temperature. After completion of the adsorption the tubes were centrifuged and the supernatant was analyzed with HPLC-SEC.

## Results and Discussion

Silica is one of the most widely used stationary phases in the laboratory practice. Without modification it is used in adsorption chromatography. For this purpose a material of 60 Å pore size is typically applied. Spherical silica with larger pores, with diameters of up to 4000 Å, is also commercially available. Such a material seemed to be suitable for purification of polysaccharides by means of SEC because the hydrodynamic radius of polysaccharides with MW of 10–1000 kDa such as alginate, chitosan, pulluan, galactomannan, heparin and methyl cellulose, calculated from the Stokes–Einstein equation based on dynamic light scattering experiments is in the range of 70–340 Å [34]. Literature data [28–30, 35] showed that bare and modified silica of larger pores (100–300 Å) can be used for SEC of polysaccharides in aqueous mode.

In this work we wanted to investigate how silica compare with more commonly used stationary phases especially from the point of view of routine laboratory separation of polysaccharides from post-synthetic impurities as well as into fractions of desired MW. Such tasks often require expensive HPLC columns or other costly resources and cheap and time-efficient alternative would be an attractive option.

**Fig. 1:**
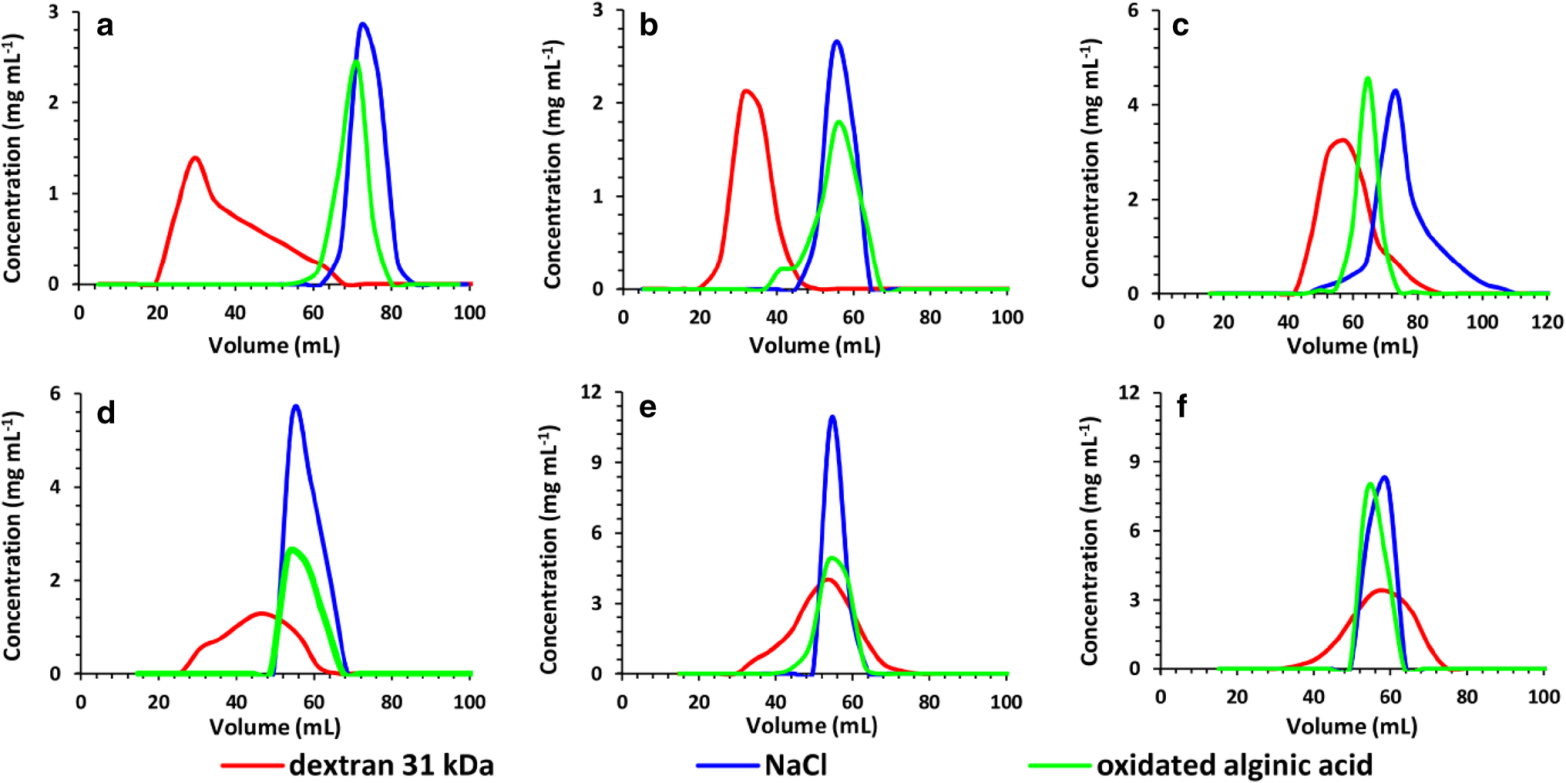
Separation of dextran and oxidized alginic acid under SEC conditions with different stationary phases. Column size 20 × 250 mm, eluate—water 5 mL min^−1^ (Sephadex 0.5 mL min^−1^) **a** Sephadex, **b** Biogel, **c** SiO_2_ 60 Å, **d** SiO_2_ 100 Å, **e** SiO_2_ 500 Å, **f** SiO_2_ 1000 Å. Portion of each substance = 75 mg in 1 mL

The evaluation of suitability of bare silica as the SEC stationary phase was performed with dextran and oxidized alginic acid as models of polymers and NaCl as a model impurity. Water as an eluent was chosen to diminish the adsorption properties of silica. Six stationary phases were investigated—SiO_2_ of 60, 300, 500, 1000 Å, Sephadex and Biogel for comparison. A constant bed size of 20 mm in diameter and 25 cm in height was used, which required different amounts of dry material. Portions of 5 mL of eluates were collected and analyzed with HPLC-SEC. The concentration of all investigated substances in each eluted fractions were determined based on external calibration lines established using standard solutions. The data were presented as chromatograms and are collected in Fig. 1. In the case of dextran it is visible that SiO_2_ of 60 and 300 Å pore size displays size-exclusion properties and it allowed to separate the polymer from NaCl. The separation efficiencies were lower comparing with Biogel or Sephadex. In the case of oxidized alginic acid only 60 Å SiO_2_ allowed separation from NaCl, whereas other stationary phases were not efficient. The result indicates that the interaction of carboxylic or aldehyde groups of oxidated alginic acid with the surface of macroporous silica gel may strongly affect its retention behavior.

Based on the results from Fig. 1 number of theoretical plates (*N*) was calculated in each case using the formula below:

$$N=8 \cdot \ln (2) \cdot {\left( {\frac{{{V_{\text{R}}}}}{{{W_{1/2}}}}} \right)^2},$$

where *t*_R_—retention time and *W*_1/2_—peak width in the middle of the height. The highest number of theoretical plates were recorded for the 60 Å SiO_2_-87, and the lower for Sephadex-16. The numbers are low, but in acceptable range for low-pressure LC.

**Fig. 2:**
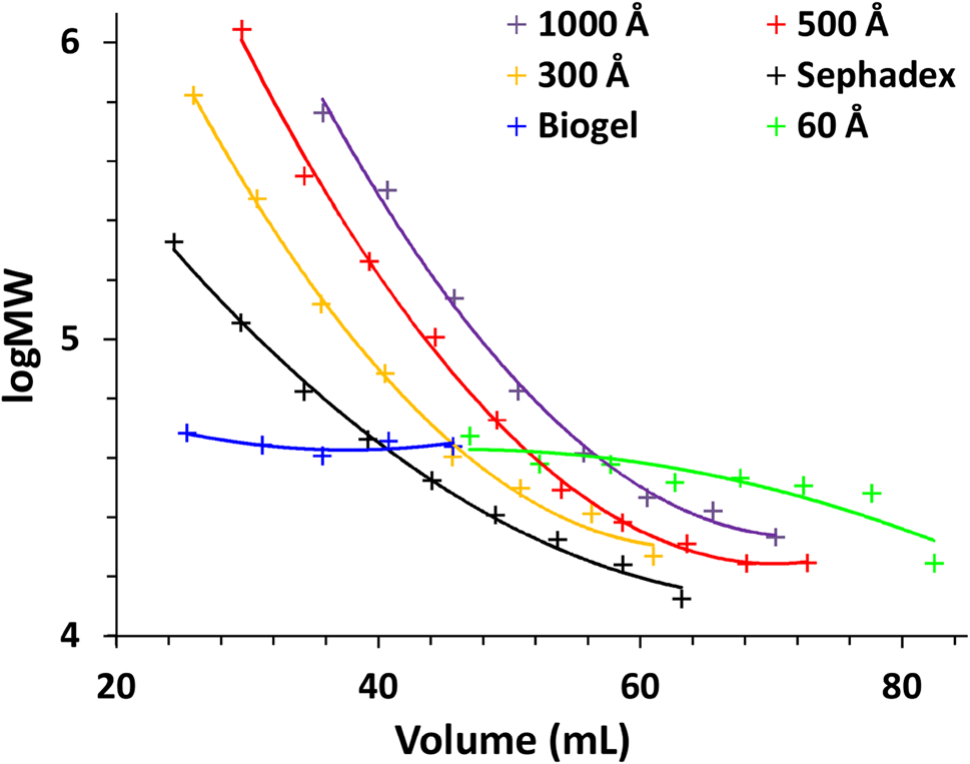
The dependence of retention volume on MW of dextrans. Column size 20 × 250 mm, eluate—water 5 mL min^−1^ (Sephadex 0.5 mL min^−1^)

Other important aspect of separation was the ability of each stationary phase to separate investigated polymers into fractions of varied MW. In the case of dextrans the HPLC-SEC Waters Hydrogel 2000 was calibrated using narrow molar mass dispersity dextran standards, which allowed to indirectly determine MW of each fraction of dextrans obtained with investigated stationary phases. With a Waters Ultrahydrogel 2000 column it was impossible to determine the MW of alginic acid fractions because the retention time was strongly affected by the injected amount. The results are shown in Fig. 2. Despite the ability to separate dextran from NaCl Biogel and SiO_2_ 60 Å did not separate dextran into fractions, whereas other phases allowed fractioning of dextran by means of MW. For example, it was possible to easily obtain fractions of an average MW = 720 kDa (logMW 5.8) and 25 kDa (logMW 4.3) using SiO_2_ 300 Å. It is worth mentioning that dextrans may have branches of smaller chains linked to the backbone and the separation is not necessarily based only on the molar mass for all investigated phases.

**Fig. 3:**
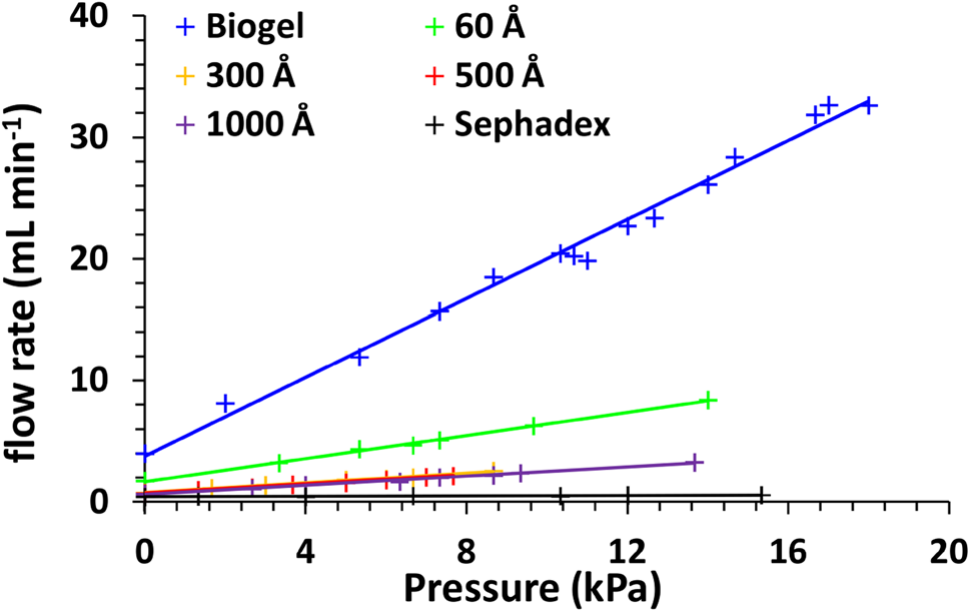
The effect of pressure on the flow rate of water. Column size 20 × 250 mm

Based on this result it can be concluded that two stationary phases are the most attractive for separation—Sephadex and silica of 300 Å. In the former case a serious drawback was a high back pressure and low flow rates of the mobile phase. The possibility of stimulation of mobile phase flow by external pressure was investigated. The results are presented in Fig. 3. In the case of Sephadex the increase of the air pressure gave very little increase in the flow rate, which was approximately 0.5 mL min^−1^. Therefore, the entire process of separation was slower comparing with SiO_2_ of any pore size, where it can be increased up to 3–8 mL min^−1^ by increasing the air pressure. Biogel showed very little flow resistance. Comparing with Sephadex the use of SiO_2_ is more time-efficient as the same sample can be purified more times or a longer column could be used maintaining acceptable flow rates. No significant changes of SEC properties were observed at different flow rates in investigated range (Fig. 3) for SiO_2_.

**Fig. 4:**
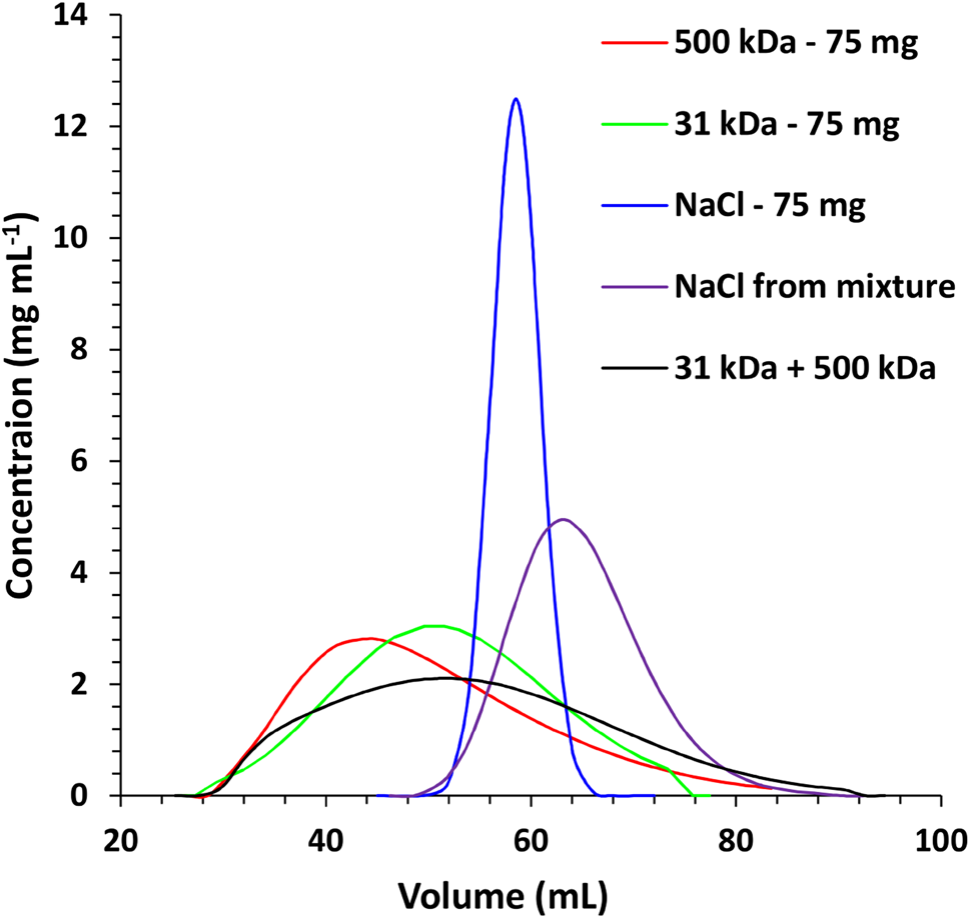
Separation of 500, 31 kDa dextrans and NaCl under SEC conditions. Column size 20 × 250 mm, eluate—water 5 mL min^−1^. Stationary phase SiO_2_ 300 Å

**Fig. 5:**
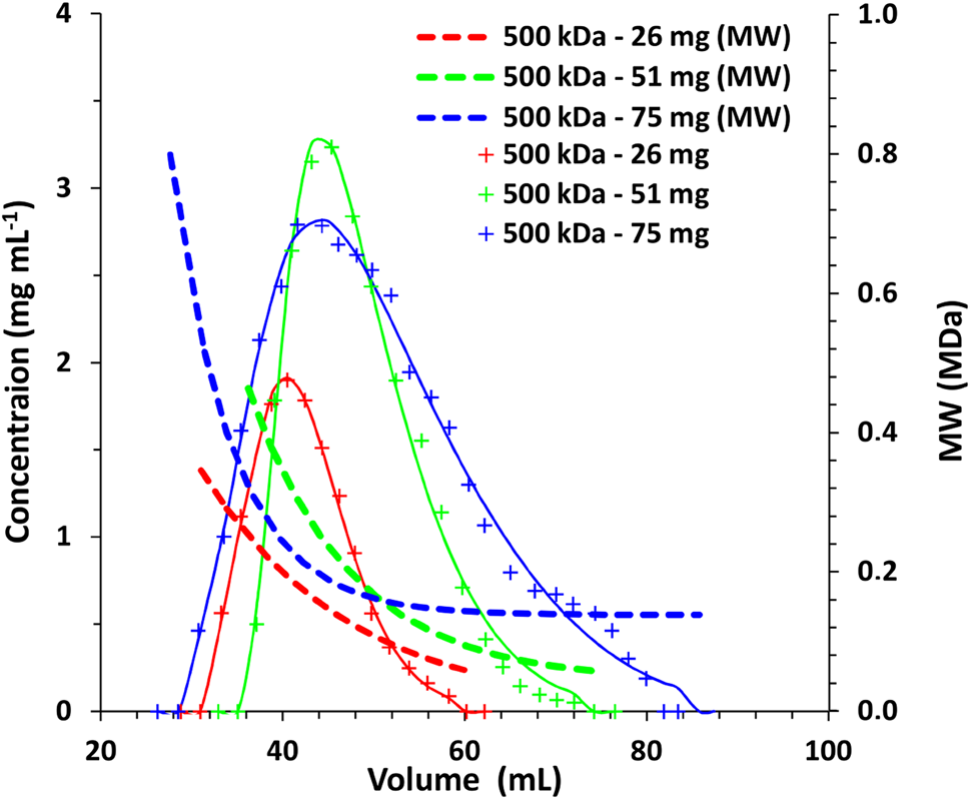
Effect of the sample amount on the resolution of the SEC column. SiO_2_ 300 Å, size 20 × 250 mm, eluate—water 5 mL min^−1^

Because the middle size silica (300 Å) showed the best properties if dextran was considered, it was tested as a SEC stationary phase in separation of a three-component mixture containing two dextrans (31 and 500 kDa) and NaCl. Such a test was aimed at separation of a typical mixture obtained from natural sources after preliminary separation by means of precipitation. The results are shown in Figs. 4 and 5. It is clear that the separation of dextran from salt is at least partially possible even at high loading. In general fractions of MW > 200 kDa should not be contaminated while fractions of MW lower than 50 kDa might contain some NaCl especially when high amount of NaCl is present. The difference between the average MW of dextran in a portion of 2 mL eluate varies with the fraction number. Higher fractions of MW of approximately 300 kDa differ by 50–100 kDa, whereas lower fractions of MW of 50 kDa only by 10–25 kDa. The effect is slightly dependent on the amount of the sample, but even at high loading, when peaks are broadened, it is still possible to separate fractions that differ in MW by 50–100 kDa (Fig. 5). From Figs. 4 and 5 it is also visible that the high total amount of the sample results in broadening of all the peaks, especially NaCl, which alone in a portion of 75 mg elutes as a narrow peak, whereas in a mixture with polysaccharides (150 mg) it elutes as a broad peak. The results indicate that the total amount of the separated mixture can reach 225 mg (in 0.5 mL) for a 20 × 250 mm column. If possible salt presence should be avoided when fractioning of polysaccharide is the primary task.

Based on the data shown in Figs. 1 and 2 and the equation [36]:

$${R_{\text{s}}}=\frac{{{V_{{{\text{R}}_{\text{2}}}}} - {V_{{{\text{R}}_{\text{1}}}}}}}{{2\left( {{\sigma _1}+{\sigma _2}} \right)}} \approx \frac{{\Delta {V_{\text{R}}}}}{{4\sigma }}$$

it was calculated that the minimal difference of MW of dextrans that can be separated when the desired resolution *R*_s_ = 1 is in the range of 200–300 kDa. Average peak standard deviation (*σ* = 2.5 mL) was calculated from Fig. 1 by fitting Gaussian curves while retention volumes of 35–45 mL were assumed to calculate the difference of MW from Fig. 2.

To investigate the potential use of macroporous silica for separation of other water-soluble polymers a series of PEGs were investigated. An attempt to separate PEGs of MW of 2–20 kDa in the same manner as dextrans failed as they were not eluted from the SiO_2_ column regardless of the sample amount. The results were in agreement with the literature report concerning adsorption of PEGs by silica [25].

**Fig. 6:**
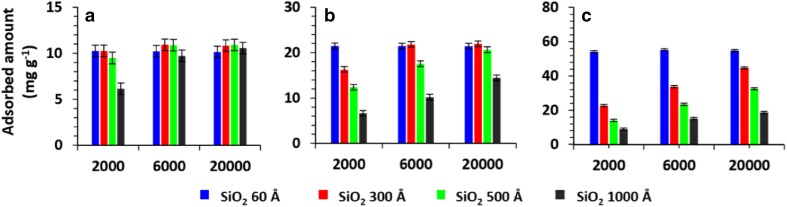
The amount of adsorbed PEGs on SiO_2_ of various pore diameters towards PEG 2, 6 and 20 kDa. Amount of SiO_2_ 200 mg, volume of PEG solution 1.5 mL, time of mixing 30 min. **a** 1.5 mg mL^−1^, **b** 3.0 mg mL^−1^, **c** 7.5 mg mL^−1^. Error bars represent the standard uncertainty

To investigate the amount of PEGs adsorbed on SiO_2_ of different pore size the solutions of three model PEGs and one dextran (31 kDa) were mixed with SiO_2_ and intensely vortexed for 30 min. The concentration of PEG in the supernatant was determined by HPLC-SEC and the amount adsorbed was calculated. The results are summarized in Fig. 6.

The results showed that the amount of adsorbed PEGs increases with the decrease of the pore size and is not significantly affected by MW of the polymer. A higher amount of PEG is adsorbed when a more concentrated solution is used. The process is not reversible, that is the corresponding amount of PEG was not washed out from the bed when pure water was added to saturated silica. The latter finding explains the lack of elution from the column observed earlier. For comparison, the amount of reversibly adsorbed dextrans (31 kDa) on silica was less than 1 mg mL^−1^.

## Conclusion

A possibility of application of bare silica of pores diameter in the range of 60–1000 Å as a SEC stationary phase was investigated. It was shown that 300 Å SiO_2_ is the most suitable for separation of dextrans of MWs of 10–1000 kDa from small molecules such as NaCl and it is also capable of fractioning of the polymer into fractions of different MWs. Comparing with standard stationary phases routinely used, such as Sephadex or Biogel, silica is less efficient if purification from salt is considered. However, the use of silica is less time consuming comparing with Sephadex and silica is considerably cheaper than both Sephadex or Biogel. 60 Å silica is capable of separation of oxidized alginic acid from NaCl, which is impossible with either Sephadex or Biogel. It indicates that 60 Å silica stationary phase can be used for purification of some polysaccharides (presumably only negatively charged) from salts or other small molecules. All investigated silicas also showed strong affinity towards PEG and were not useful as a stationary phase for chromatographic separation of PEGs. Comparison between investigated polymers lead to a conclusion that only separation of dextran using macroporous silica fit the typical SEC model. Presented stationary phases may especially find routine application in laboratories dealing with polysaccharides and their purification as well as for larger scale purification when price of the stationary phase is an issue. The results can also be useful during selection of stationary phase for various polysaccharide-related preparative and analytical tasks.
